# Translation and psychometric evaluation of the Persian version of “good death inventory- short Form” from the perspective of family-members of cancer patients

**DOI:** 10.1186/s40359-023-01305-0

**Published:** 2023-09-02

**Authors:** Hosein Mohammadi Roshan, Abbas Ebadi, Leila Karimi, Salman Barasteh

**Affiliations:** 1 Student Research Committee, Baqiyatallah University of Medical Sciences, Tehran, Iran; 2 Behavioral Sciences Research Center, Life Style Institute, Baqiyatallah University of Medical Sciences, Tehran, Iran; 3 Nursing Faculty, Baqiyatallah University of Medical Sciences, Tehran, Iran; 4 Behavioral Sciences Research Center, Life Style Institute, Nursing Faculty, Baqiyatallah University of Medical Sciences, Tehran, Iran; 5 Health Management Research Center, Baqiyatallah University of Medical Sciences, Tehran, Iran

**Keywords:** Cancer, End of Life Care, Good death, Hospice, Palliative Care, Quality of dying and death, Validation, Inventory

## Abstract

**Introduction:**

Achieving good death is among the objectives of palliative care in patients with cancer. There should be an instrument for evaluating the quality of palliative care provided by family members at the end of life. This study was done to assess the psychometric properties of good death inventory- short form according to the perspective of family of patients with cancer.

**Method:**

This methodological study was done in 2022 at two hospitals in Tehran. The translation was done via forward-backward method. Face validity was examined through cognitive interviewing with 10 family members. The content validation, were used by assessment the opinions of 10 palliative care specialists. The construct validity was explored through exploratory factor analysis and examination of convergent validation with care evaluation scale 2.0, as well as inspection of correlation by answering two general questions of satisfaction with treatment and end of life quality of life. The scale’s reliability, internal consistency was calculated using Cronbach’s alpha coefficient and stability via test-retest.

**Results:**

Overall, 204 family members of patients with cancer were included. In the exploratory factor analysis, three factors of peace, hope, and value as well as quality of care were extracted with cumulative variance of 41.8%.A significant and suitable correlation between the total scores of the participants Good death inventory-short form and care evaluation scale2.0 (r = 0.459, P < 0.001) and general satisfaction with end-of-life care (r = 0.423, p < 0.001) as well as the patient’s general quality of life (r = 0.539, p < 0.001). The Cronbach’s alpha coefficient for the questionnaire was found 0.842, and the stability was confirmed with Intra cluster correlation coefficient = 0.851.

**Conclusion:**

the Persian version of good death inventory-short form is a valid and reliable questionnaire which can investigate the factors associated with good death according to patients’ family members’ perspective.

## Introduction

Cancer is the second cause of mortality worldwide [1] and the third cause in Iran [2]. In 2018, around 9.6 million people died because of cancer, being around one out of every six deaths worldwide [1]. Annually, 90,000 new cases of cancer are recorded in the country [2]. According to World Health Organization (WHO), by 2030, the morality caused by cancer would reach 13 million [3]. Most patients with cancer at the end of life face concerns related to their family and friends, socioeconomic, self-associated, healthcare team and treatment process at hospital, and or religious-spiritual concerns. In case these concerns and needs remain neglected, the patient’s welfare as well as quality of life and quality of death would be impaired [4].

Good death is an important goal and outcome for evaluating the end-of-life care for patients with cancer [5]. Due to differences in the view of patients, care providers, acquaintances, and healthcare providers, various definitions have been presented for good death [6]. Some factors affecting good death can include controlling pain and symptoms, decision-making on treatment preferences, sense of termination, being seen, and understood as a person, preparation for death, presence of family, past experiences of others’ death, death in the favorite place, having a good relationship with the family or healthcare staff, sense of not being a burden, culture, financial issues, religious and spiritual peace, independence, age, as well as psychological and social support [6–13].

there should be an instrument to evaluate the end-of-life care by the companions to provide a comprehensive palliative care [14]. Under such conditions, researchers take one of the following two measures: designing a new questionnaire which is time-consuming and requires observation of special scientific principles, and use of the current (foreign) questionnaires, whose reliability and validity have already been confirmed [15].

So far, various instruments have been presented for investigating good death and quality of end-of-life care, which include Quality of Dying and Death (QODD) [16], Good Death Inventory (GDI) [12], Quality Care Questionnaire – End of Life (QCQ-EOL) [17], and Care Evaluation Scale (CES) [18].

One of the instruments for exploring good death in patients with cancer is the Good Death Inventory. This instrument was designed by Miyashita et al. in 2008 for examining the factors associated with good death in patients with cancer [12]. Its short form includes 18 items in ten primary areas and eight secondary areas, which is completed by the family members of patients with cancer. The sum of scores ranges from 18 to 126, with higher scores indicating greater probability of achieving a good death [12]. So far, the reliability and validity of this questionnaire have been examined in Korean [19], Chinese [5], and Spanish [20].

Review of the literature shows sparsity of studies on good death in Iran. Also, so far there has been no instrument for exploring the factors associated with good death according to patients’ family views, where the patients and society need to specialized end-of-life services [21] highlights the importance of conducting further studies in this regard. Thus, this study was done to assess the psychometric properties of the Good Death Inventory- Short Form according to the family of patients with cancer in Tehran.

## Method

### Study design

The present methodological research has dealt with Persian translation and assessment of the psychometric properties of Good Death Inventory- Short Form.

### Study population/sampling

The population of this study consisted of family members of patients with cancer who had died either in Baghiatallah or Shohaday-E Tajrish hospitals. Sampling of the present study was done in 2022. The inclusion criteria were; definite diagnosis of cancer in the deceased, the deceased being at least 20 years of age, the family member’s willingness to participate in the study, reading and writing in Persian, the family member awareness of the malignancy diagnosis, and possibility of establishing communication with the mourning family members through SMS or common foreign social media platforms (WhatsApp, Telegram) or Iranian platforms (Eitaa and Soroush). The exclusion criteria included lack of willingness to continue cooperation by the family members in completing the questionnaires, or incomplete filling of questionnaires, participation of the subjects in other studies with themes of good death, and psychological disorders of the participants.

### Demographic information questionnaire

The demographic information related to the patient included age, gender, marital status, type of cancer, level of education, and income. The information related to the member included relation to the patient, frequency of care provision for the patients, and level of education. Also, the opinions of the family members regarding overall satisfaction with the treatment and general satisfaction with quality of life of their patient at the end of life were examined.

### Good death inventory- short form

The GDI was designed by Miyashita et al. in 2008. The short form of this inventory includes 18 items in 10 main and 8 secondary domains.

The ten main areas, were including physical comfort, dying in a favorite place, maintaining hope and pleasure, having good relationship with medical staff, not being burden, having good relationship with family, independence, environmental comfort, being respected as a person, end of life and eight sub-domains were receiving adequate treatment, natural death, preparation for death, future control, ignorance of death, pride and beauty, feeling that one’s life is worth living, religious and spiritual comfort which would be completed by the family members of patients with cancer with seven-point Likert scale (1 = absolutely disagree, 2 = disagree, 3 = disagree to some extent, 4 = uncertain, 5 = agree to some extent, 6 = agree, and 7 = absolutely agree). They were categorized in four factors named physical and psychological comfort, decision making and relation to medical staff, family relationship, and psycho-existential issues. The instrument reliability was assessed by test-retest method (ICC = 0.52), and Cronbach alpha method (0.74–0.95) [12].

### Care evaluation scale version 2.0 (CES2.0)

 [18]CES2.0 was developed in 2017 to remove the problem of wrong answers in the original scale by Miyashita et al. It includes 10 items which would be completed by the mourning families with six-point Likert scale. Higher scores indicate good care process or structure (22).

### Translation procedure

Forward-backward translation was done according to the standard protocol of the World Health Organization [22]. In the forward translation, the original English version of the inventory, after acquiring permission from its developer, was translated to Persian. In the backward translation stage, the Persian translation was retranslated back to English by two natives with mastery over Persian and English who had not awareness of the original version, whereby an English version was obtained. The two retranslated English versions obtained in the previous stage were sent to the scale developer and confirmed. Next, cultural adaptation and other psychometric properties were done as follows.

### Procedure

The questionnaire had been designed online. Overall, there were 772 subjects whose patient had died between one month and one and year and half before initiating the research. The subjects were contacted, with 399 of them being responsive. The link of online questionnaire including demographic information, GDI-short form, CES2.0, and questions of general satisfaction with end-of-life care and quality of life was sent to them. Ultimately, 204 (51%) subjects completed the online questionnaire.

### Face validity

After completion of the translation procedure, cognitive interviews were done for exploring the qualitative face validity. Also, in order to understand the phrases and words, the optimal fit of the items, the possibility of ambiguity in the phrases or the existence of insufficiency in the meanings of the words, a cognitive interview is conducted with the target group [23, 24]. Accordingly, interview was done with 10 family members, who were different in terms of socioeconomic and education level. They were asked to evaluate the legibility, clarity, and structure of items, ease of understanding, confusing words, classification of items, ease of answering the items, linguistic forms, and word arrangements [25].

### Content validity

The content validation was done to explore all important aspects of the intended concept of the instrument as well as acceptance of execution and totality of the instrument by experts [23]. To examine the content validity, the Persian version of the inventory was given to 10 specialists in palliative care and they were asked to examine the relevancy of the items through four-point Likert scale. Ultimately, CVI score was calculated for the items. A content validity index higher than 0.79 is considered suitable, 0.7–0.79 needs correction and revision, and score below 0.7 is unacceptable, and should be omitted [26]. To examine the ceiling and floor effects, the samples that had been taken for construct validity were used. When more than 15% of participants acquire the maximum or minimum achievable score, it is called ceiling and floor effect [27]. Existence of ceiling and floor effect indicates insufficient content validity.

### Item analysis

The items were analyzed with the aim of checking the initial reliability. The effect of each item on the reliability and the identification of problematic and incorrect items and their correction were investigated. At this stage, the final and modified version of the inventory was given to 30 participants. Using SPSS 26 software and loop technique, the correlation between the items and the correlation of each item with the total score were measured. Cronbach’s alpha was also described after removing each item [27].

### Construct validity

To explore the construct validity of this scale, exploratory factor analysis (EFA) and convergent validity methods were used.

### Exploratory factor analysis (EFA)

Exploratory factor analysis (EFA) is used for examining the underlying structure of a relatively large set of variables. The minimum sample size required for EFA is 3–10 participants per each item [28]. To examine the EFA, 204 family members of patients with study cancer were included in the through available sampling. To check the adequacy of the sampling and the suitability of the subjects, Keiser-Meyer-Olkin (KMO) and Bartlett’s test were done. A KMO value closer to 1 is more suitable for factor analysis; however, generally a score larger than 0.5 is acceptable, and is more suitable at greater than 0.7 [27]. The Bartlett’s test with significance level blow 0.05 is acceptable [29, 30]. Suitable results of KMO and Bartlett’s test indicate existence of desirable correlation matrix for factor analysis [23]. The value of factor load is the relationship between each factor and each item of questionnaire. In order for each item to remain, its relationship should be suitable. The minimum factor load in this study was considered 0.3. In case of factor load lower than 0.3, the relationship between the factor and item is weak [31, 32]. For extraction of factors, based on indices of skewness (± 3) and kurtosis (± 7), the maximum likelihood (ML) method, and for interpretability of the factors, varimax rotation was used [33].

### Convergent validation

To examine the convergent validation, the respondents concurrently to both the Persian version of the GDI and theCES Version 2 [34]. The correlation between the GDI- Short Form and CES2.0 was measured via Pearson correlation coefficient [35]. Also, the respondents responded to two general questions of satisfaction with end-of-life care and terminal quality of life, and their correlation with the GDI was explored.

### Reliability

To determine the reliability of the Good Death Inventory- Short Form, two methods of internal consistency and stability were examined. To measure the internal consistency of the instrument, Cronbach’s alpha coefficient was calculated. To have good and sufficient internal consistency, the Cronbach’s alpha coefficient should be greater than or equal to 0.7 [36]. To determine the stability of the instrument, test-retest method was used with sample size of 30 subjects. In this study, the retest interval was considered 14 days. The scores acquired at these two stages were compared via Intra-cluster correlation (ICC) index. ICC index above 0.80 is assumed as desired stability [37]. In this research, the total-item correlation was also inspected. The correlation between each item and the total score of the scale was calculated. Next, based on these correlations, decisions were made on keeping or discarding the items, whereby the items with correlation lower than 0.3 were removed [36].

In this study, standard error of measurement was also calculated. Small SEM of the instrument is important since changes above it are clinically important. The SEM is calculated for quantifying the accuracy of score of each person. To calculate the instrument’s SEM, the following formula can be used. In this formula, SD (standard deviation) reflects the sum of the two test and retest samples [38].

$$SEM=SD\times \sqrt{(1}-ICC)$$

### Ethical considerations

The permission of study was taken from Baghiatallah University of Medical Sciences with the ethics code: IR.BMSU.REC.1400.122. After acquiring written permission from the instrument’s developer through Email, the process of translation was initiated. Before starting the research, the participants were informed about the research objectives.

### Data analysis

SPSS 26 was used for data analysis. In all analysis, p < 0.05 was considered significant.

## Results

### Sociodemographic and clinical status

#### Participants’ characteristics

The deceased patients included 108 men and 96 women with the mean age of 63.54 years. Most patients at the time of death were married (82.8%). Most of them had average level of income (60.3%) and 38.7% also reported low household income. The type of cancer and level of education are reported in Table 1.

**Table 1:** Demographic information of patients

Variable	n	%	Mean (SD)
Gender			
Male	108	53	65.25(18.32)
Female	96	47	65.75(15.24)
Age			
60>	70	35	66.18(15.83)
60–70	67	32.5	65.88(16.35)
70<	67	32.5	64.35(18.64)
Marital			
Divorced	4	2	56.4(20.28)
Single	10	4.9	66.68(16.57)
Death Of Wife	21	10.3	49.5(13.79)
Married	169	82.8	63.19(16.39)
Education			
Illiterate	36	17.6	73(13.81)
Elementary	48	23.5	65.6(16.72)
Middle School	23	11.3	64.69(16.28)
Diploma	59	28.9	64.93(16.52)
University Education	38	18.6	59.55(18.84)
Income			
High	2	1	89(4.24)
Moderate	123	60.3	66.95(16.12)
Low	79	38.7	62.95(17.62)
Cancer			
Liver	18	8.8	71.38(15.68)
Lung	25	12.3	65.2(14.47)
Brain	19	9.3	62.36(21.74)
Colorectal	9	4.4	63.11(17.48)
Breast	24	11.8	66.04(14.98)
Digestion	35	17.2	70.62(13.04)
Genital	8	3.9	67(17.7)
Leukemia	14	6.9	63.57(15.91)
Prostate	12	5.9	61.16(23.92)
Other	40	19.6	61.85(17.9)

### Family member characteristics

The participants included 204 patient companions including 115 (56.4%) men and 89 (43.6%) women, with the mean age of 45.6 ± 11.87. Most questionnaire respondents were male (56.4%) and the child of the deceased person (61.8%). Most of the respondents offered daily care for their patient (Table 2).

**Table 2:** Demographic information of patients’ families

Variable	n	%	Mean (SD)
Relationship with the Patient			
Spouse	48	23.5	69.02(14.05)
Parents	3	1.5	62.33(5.5)
Child	126	61.8	64.9(16.79)
Other	27	13.2	62.25(21.88)
Family Members’ Gender			
Male	115	56.4	67.8(16.27)
Female	89	43.6	62.94(17.32)
Number of Times Patient Care			
Everyday	146	71.6	64.04(16.7)
4–6 Days A Week	24	11.8	69.66(15.93)
1–3 Days A Week	26	12.7	70.38(16.78)
Less Than One Day A Week	8	3.9	63.37(21.65)
Family Members’ Education			
Elementary	6	2.9	72.66(18.14)
Middle School	17	8.3	68.94(14.13)
Diploma	62	30.4	70.88(16.46)
University Education	119	58.3	61.81(16.65)

### Face and content validity

The face validity was confirmed using 10 family member. The items did not change in the face validity examination due to simplicity and clarity. The content validity was confirmed using opinion of 10 palliative care experts. The content validity ratio (CVI) was calculated for the items, with all items showing a score above 0.79, and none of them was eliminated at this stage. Also, the ceiling and floor effect each was calculated 0.5, indicating that the content of the designed instrument is suitable for examining the factors associated with good death.

### Item analysis

Cronbach’s alpha coefficient in the item analysis was 0.710. Also, items 2, 12, 13, 14, 15, 18 had a correlation of less than 0.3 with the total score. According to the discretion of the research team, for to maintaining the dimensions of the inventory, all the items were kept and the changes in the items were made in the joint research committee.

### Construct validity

**Fig. 1:**
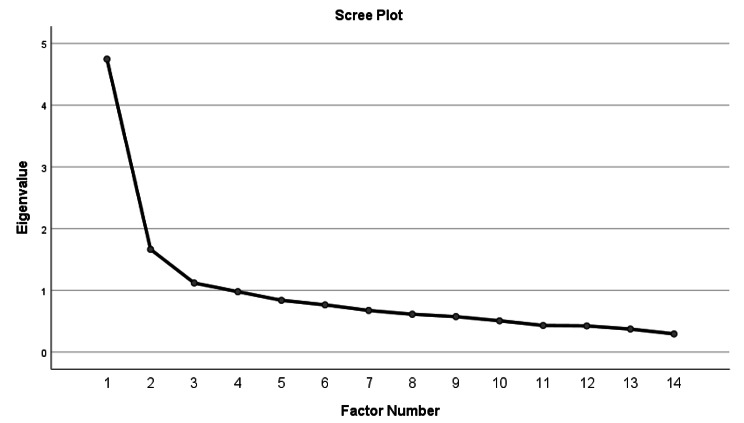
Scree plot

A KMO value of 0.833 was found, and Bartlett’s test of sphericity was significant (X2 = 852.496, df = 91, p = 0.000). Three factors were extracted and named (Table 3). These three factors were: peace (15.52%), hope and value (13.65%), and quality of treatment (12.62). At this stage, four items were removed from the inventory (Table 3; Fig. 1). Also, we found a moderate correlation between the total scores of the participants Good death inventory-short version and CES2.0 (r = 0.459, P < 0.001) and general satisfaction with end-of-life care (r = 0.423, p < 0.001) as well as correlation with general quality of life of the patient at the end of life (r = 0.539, p < 0.001).

**Table 3:** Exploratory Factor Analysis of the Persian Version of GDI-short form

Factor	Items	Factor Loading%	Variance
Factor1	Q3	0.496	15.52
	Q4	0.410	
	Q5	0.456	
	Q6	0.309	
	Q8	0.381	
	Q9	0.382	
Factor2	Q1	0.427	13.65
	Q2	0.312	
	Q10	0.361	
	Q11	0.355	
	Q18	0.394	
Factor3	Q7	0.423	12.62
	Q12	0.408	
	Q17	0.374	
Cumulative%			41.80

### Reliability

The internal consistency was obtained as 0.842 using Cronbach’s alpha coefficient. Also, the ICC was reported 0.851. The SEM was also calculated and presented in Table 4.

**Table 4:** Reliability by the method of internal consistency and relative and absolute stability

Factor	Items	Alpha	ICC	CI (95%)	M(SD)	SEM
Peace	3,4,5,6,8,9	0.775	0.859	0.704–0.93	26.8(7.15)	2.7
Hope and Value	1,2,10,11,18	0.714	0.824	0.6–0.92	25.5(5.46)	2.29
Quality of Treatment	7,12,17	0.7	0.787	0.56–0.89	14.3(4.47)	2.06
Total	1,2,3,4,5,6,7,8,9,10,11,12,17,18	0.842	0.851	0.69–0.92	66.7(13.57)	5.23

## Discussion

In the present study psychometric properties of the Persian version of Good Death Inventory– Short form assessed by face, content and construct validity through EFA and convergent validity as well as reliability by internal consistency and test-retest.

The translation process was performed carefully until reaching a final Persian version. Investigation of the face validity of the instrument using opinions of 10 family caregivers showed that the items are simple and clear. The face validity in the original Japanese version has been done by two physicians, two nurses, and two normal individuals. After a general review of the literature, the content validation has been done among researchers [12].

In this study, the content validity has been examined using opinions of 10 experts including physicians and nurses in palliative care and based on CVI calculation, whereby all items had a score above 0.79. In the study by Zhao et al., they examined and conformed the content validity of a preliminary questionnaire by a committee consisting of a nursing education specialist, a public health management specialist, an oncology nurse specialist, a clinical nursing manager, an English medical specialist, and a rehabilitation medical specialist [5].

In the present study, in the exploratory factor analysis (EFA), three factors with cumulative variance of 41.80% were extracted, which differs from the original study, where four factors have been extracted (physical and psychological comfort, decision making and relation to medical staff, family relationship, and psycho-existential issues) [12]. Nevertheless, the general framework of the questionnaire seems to have been preserved, since in the present study, the factors of family relationship and psycho-existential issues have been accumulated in to hope and value factor.

in the study by Miyashita et al., the criterion validity was explored through concurrent use of CES and examination of the general satisfaction of the respondents, where all items have had correlation with the total score of CES (r = 0.26) [12].

In this study, the total score of the GDI had a correlation with the general satisfaction with end-of-life care (r = 0.423) and with general quality of life of patient at the end of life (r = 0.539). Based on these findings, in the study by Miyashita et al., again the GDI correlation with the general satisfaction of participants was r = 0.39 [12]. In the study by Juanjuan Zhao et al., the total score of GDI had an average correlation with general satisfaction with the treatment (r = 0.411, p < 0.001) and with quality of life (r = 0.468, p < 0.001) as well as with general quality of death (r = 0.441, p < 0.01) [5]. In the study by Shin, Dong Wook, the correlation of the total score of GDI with quality of life at the last week was (r = 0.56, p < 0.001) and with general satisfaction with the treatment was (r = 0.44, p < 0.001) [19].

In the present study, reliability measurement of the Persian version of GDI – short form was confirmed through internal consistency (Cronbach’s alpha = 0.842) and stability (ICC = 0.851), indicating suitable stability of the instrument. In line with these findings, in the study by Miyashita et al., again alpha = 0.94 (0.74–0.95) with ICC = 0.52 [12]. Similarly, in the study by Shin, Dong Wook et al., again alpha = 0.93 (0.69–0.94) [19]. In the study by Juanjuan Zhao et al., again using internal consistency, the Cronbach’s alpha = 0.896 (0.561–0.950) [5].

Ultimately, it can be stated that the psychometric results of the GDI – Short form in the Persian version have been favorable, and this inventory can be a good scale for examining the factors associated with good death according to patient’s family members’ perspective. Other advantages of this instrument included the low number of items, and short time required for its completion. Meanwhile, this instrument is used for examining good death according to the patients’ family members’ perspective, who can be credible sources for exploring the factors associated with the end of life of their patient life. This inventory is not limited to a special disease, and it can be used in other life-threatening diseases as well as in other healthcare centers including hospital, elderly care centers, or hospices.

## Conclusion

The Persian Version of GDI-short form is a reliable and valid questionnaire which can investigate the factors associated with good death according to the patients’ family members’ perspective. Thus, this instrument can be used in clinical evaluation as well as research purposes of family members in Iranian society.

### Limitations

This study only examined the views of the family caregivers’ of cancer patients. Meanwhile, most participants consisted of women, which can affect the results. Considering the limitations of access to the Internet at the time of sampling, access to the subjects had been severely limited. Also, since the research subjects were family of patients dying of cancer, their cooperation with the researchers was challenging, and the participation rate was 51%. In this study, due to limited number of participants, confirmatory factor analysis was not done, and it is recommended to also examine the construct validity of this inventory using confirmatory factor analysis in future. Another limitation of the study was the available sampling, which suggests that random sampling should be used in the future study.

## Data Availability

All data generated or analyzed during this study are included in this published article.
